# Acute upper gastrointestinal mucosal lesions caused by COVID-19 infection: a case report

**DOI:** 10.3389/fmed.2025.1618638

**Published:** 2025-07-31

**Authors:** Ze Qi, Bing-Jie Yan, Ruo-Han Hu, Lei Li

**Affiliations:** ^1^Department of Gastroenterology, Affiliated Hospital of Shandong Second Medical University, Weifang, China; ^2^Shandong Second Medical University, Weifang, China

**Keywords:** COVID-19, COVID-19 pandemic, SARS-CoV-2 virus, ACE2, digestive system infection, acute upper gastrointestinal mucosal lesions

## Abstract

**Background:**

COVID-19 enters human cells by binding its surface protein to angiotensin-converting enzyme 2 (ACE2) present in the host. ACE2 is expressed in various organ cells in the human body. Consequently, SARS-CoV-2 can invade gastrointestinal epithelial cells through ACE2, leading to the manifestation of gastrointestinal symptoms. However, the occurrence of gastrointestinal mucosal lesions and bleeding is rare.

**Case summary:**

A 34-year-old man with no previous medical history was hospitalized due to two weeks of nausea, hematemesis, and one week of fever. Gastroscopy showed widespread inflammation and necrotic tissue in the stomach, suggesting erosive hemorrhagic gastritis. Laboratory tests confirmed COVID-19 infection and blood hypercoagulability. After treatment, the patient was discharged. Follow-up gastroscopy showed mucosal lesion healing after recovery from COVID-19.

**Conclusion:**

The patient underwent endoscopy and pathological analysis. ACE2 immunohistochemistry was performed on the pathological tissues to investigate the cause of the lesions. After ruling out other possible factors, the final results indicated that the patient’s gastric mucosal lesion was caused by COVID-19 infection. This suggests that further research on COVID-19 should consider its gastrointestinal symptoms and diseases.

## Introduction

COVID-19 (coronavirus disease 2019), caused by the SARS-CoV-2 virus, emerged in December 2019 and rapidly escalated into a global pandemic, prompting the World Health Organization to declare a global health emergency on March 11, 2020 (1). This disease presents with a wide range of symptoms, primarily respiratory manifestations such as fever and cough, with approximately 80% of cases being mild. However, about 20% of patients may develop severe illness and 5% progress to critical conditions like pneumonia or acute respiratory distress syndrome, often requiring mechanical ventilation and intensive care (2). Patients with underlying comorbidities—including hypertension, diabetes, and cardiac or renal diseases—face a higher risk of severe complications such as septic shock, acute respiratory distress syndrome, and death (3). Recent research and clinical data indicate that gastrointestinal symptoms are relatively common. Diarrhea is the most prevalent gastrointestinal symptom, and nausea, vomiting, and loss of appetite are also frequently reported (4). However, reports of acute gastrointestinal mucosal injury as the initial presentation of COVID-19 are relatively uncommon.

SARS-CoV-2 enters cells through the ACE2 receptor, which is activated by the intracellular serine protease TMPRSS2 (5). Research has demonstrated that this receptor is expressed not only in the lungs but also in extra-pulmonary organs, including the gastrointestinal tract, leading to the gastrointestinal symptoms associated with COVID-19 (6). Reports indicate that common gastrointestinal symptoms among COVID-19 patients include diarrhea, nausea, vomiting, and abdominal discomfort. Gastrointestinal bleeding resulting from COVID-19 infection is relatively uncommon, and there is limited literature available on endoscopic observations of gastrointestinal lesions. We analyzed multiple gastroscopy results from the patient described in this study and conducted ACE2 immunohistochemical staining on the biopsy samples. This analysis aimed to investigate the presence of ACE2 expression at the lesion site and its expression tendency during disease progression.

## Case report

**Figure 1:**
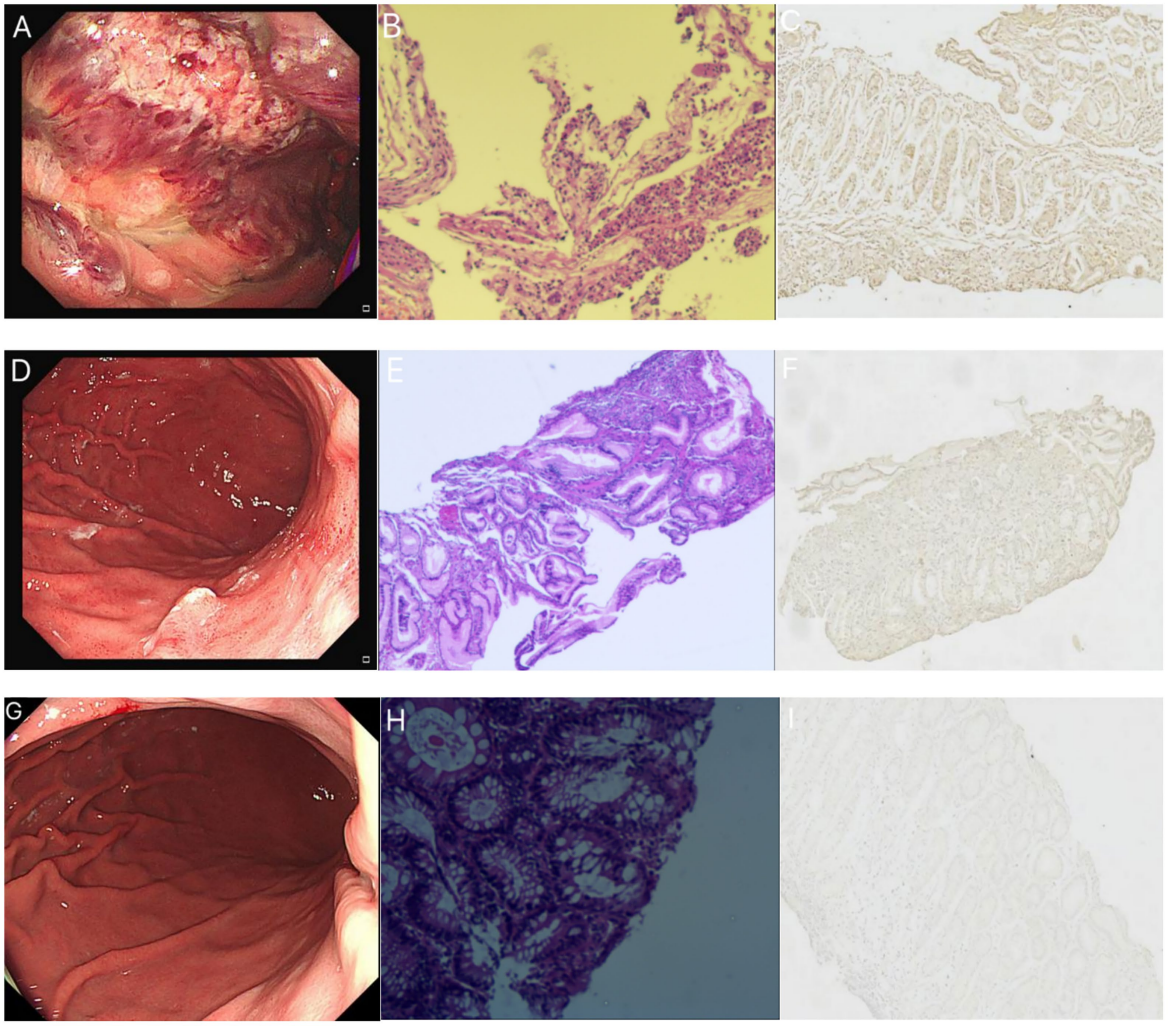
The patient’s gastroscopic findings, pathological HE staining, and ACE2 immunohistochemistry results. **(A–C)** December 21, 2022. **(A)** The patient’s gastroscopy revealed a rough mucosa with erosion and bleeding in the fundus and body. **(B)** HE staining demonstrates inflammatory exudates, necrotic tissue, acute and chronic inflammation of the mucous membranes surrounding the necrotic tissue, and lymphocyte infiltration in the intraepithelial interstitium. **(C)** ACE2 immunohistochemical staining of the biopsy tissue showed bright brownish yellow color, indicating strong positive. **(D–F)** January 16, 2023. **(D)** The patient’s gastroscopy revealed a 0.4 cm diameter convex lesion on the posterior wall of the upper gastric body. The surface exhibited erosion and was covered with white mucus. **(E)** HE staining demonstrated chronic mucosal inflammation, focal neutrophil and lymphocyte infiltration, small blood vessel presence, and histiocytic hyperplasia. **(F)** The ACE2 immunohistochemical staining of biopsy tissue was weaker compared to the previous results, showing weak positive. **(G–I)** August 14, 2023. **(G)** The patient’s gastroscopy displayed a mucosal bulge in the posterior wall of the upper end of the stomach body. The bulge appeared red with residual mucosal congestion and scattered erythema. **(H)** HE staining exhibited acute and chronic mucosal inflammation with erosion. **(I)** The ACE2 immunohistochemical staining of the biopsy tissue was extremely light yellow, and the positive expression was not obvious.

A 34-year-old male with no prior history of gastrointestinal or other medical conditions was admitted to our hospital on December 21, 2022. He presented with a two-week history of nausea with episodes of hematemesis and a one-week history of fever. Two weeks ago, the patient experienced episodes of vomiting mucoid substances from the stomach, along with the presence of blood. The patient developed fever a week ago and had no other symptoms of respiratory infection. A nasopharyngeal COVID-19 polymerase chain reaction (PCR) test result was positive. The patient experienced a recurrence of nausea and vomiting 8 h prior to admission. The vomit consisted of stomach contents mixed with blood, with an estimated volume of approximately 40 mL. The chest and abdominal CT revealed a few fibrous lesions in both lungs, pulmonary bulla, and alterations in the stomach body. Gastroscopy findings indicated diffuse inflammatory exudates and necrotic tissue in the stomach, suggestive of erosive hemorrhagic gastritis (Figure 1A). Pathological examination revealed acute and chronic inflammation of the mucous membrane surrounding the necrotic tissue, along with erosion and lymphocyte infiltration in the stomach (Figure 1B). Initially, mucosa-associated lymphoid tissue lymphoma (MALT) was suspected based on endoscopic findings. However, this was later ruled out by pathological examination. Subsequent laboratory tests after admission revealed an increase in red blood cells and hemoglobin levels, along with a decrease in average hemoglobin concentration, possibly indicative of hemoconcentration following bleeding. The coagulation function test revealed hypercoagulability of blood, with a D-dimer level of 1.260 mg/L. Following hospital admission, the patient received intravenous infusion of rabeprazole 20 mg twice daily and oral Kangfuxin liquid for gastric mucosa repair, while being maintained on nil per os (NPO) status with parenteral nutrition support. For the COVID-19 infection, antiviral therapy with oral oseltamivir 75 mg twice daily was administered. The patient demonstrated symptomatic improvement after three days of treatment and was subsequently discharged. The aforementioned medications were continued orally during the post-discharge period.

The patient was readmitted to our hospital on January 15, 2023. At this time, the patient had no respiratory symptoms such as cough and fever, and The PCR test result was negative. Subsequent gastroscope reexamination revealed depressed lamellar mucosa near the gastric antrum in the lower part of the greater curvature of the stomach body, irregular glands, and reddened mucosa. A convex lesion, approximately 0.4 cm in diameter, with an eroded surface covered in white mucus, was observed on the posterior wall of the upper segment of the stomach. Hyperemia and edema were observed in the gastric antrum mucosa (Figure 1D). No other significant abnormalities were detected. The gastroscopic examination showed improvement compared to the previous examination. Biopsy of the lesions revealed chronic inflammation with acute inflammation and erosion in the mucosa near the antrum in the lower part of the greater curvature of the stomach, along with minimal eosinophilic infiltration in the interstitium. Additionally, chronic inflammation, focal neutrophil infiltration, and lymphocyte infiltration were observed in the posterior mucosa of the upper part of the stomach (Figure 1E). In August 2023, after a six-month interval, the patient underwent a follow-up gastroscopy examination, which revealed complete healing of the gastrointestinal mucosal injury (Figure 1G), with only mucosal inflammation observed on HE staining (Figure 1H).

## Materials and methods

In order to explore the correlation between the gastric mucosal lesions and COVID-19 in this patient, ACE2 immunohistochemistry was performed on the patient’s pathological tissues.

All specimens were fixed in 10% formalin solution for approximately 24 h, followed by embedding in paraffin and sectioning. The tissue sections were dewaxed and rehydrated through a series of steps with xylene and ethanol baths. Place the slides in a 0.2% Triton X-100 solution for 10 min. Then, add 3% H2O2 to the tissue and incubate at 37°C for 30 min. Next, immerse the slides in boiling EDTA solution for 1 h to retrieve antigens. Following antigen retrieval, add 10% goat serum to the tissue and incubate at room temperature for 1 h. Subsequently, add anti-ACE2 antibody (Abcam, UK) to the tissue and leave at room temperature for 1 h, followed by overnight incubation at 4°C in a humidified chamber. The next day, allow the slides to equilibrate to room temperature for 30 min, then add anti-rabbit antibody and incubate at room temperature for 1 h. After each of the above steps, wash the slides three times with PBS. Finally, cover the slides completely with DAB chromogen solution and incubate at room temperature for color development. Once positive staining is observed, rinse the slides with tap water to stop the color reaction. Counterstain the nuclei with hematoxylin and mount the slides for observation.

## Discussion

ACE2 immunohistochemical staining was conducted on three endoscopic biopsies obtained from the patient. In our study, we noticed a gradual change in the immunohistochemical findings of ACE2 in the pathological tissues, transitioning from strong positive to weak positive as the patient’s condition improved. This suggests a decrease in the positive expression of ACE2 in the pathological tissues (Figures 1C,F,I). Considering that ACE2 is recognized as a binding target for the invasion of SARS-CoV-2 into cells (7), it is speculated that this lesion is associated with COVID-19 infection.

The patient had no history or symptoms of gastrointestinal disease. The typical sites for gastric ulcers are the lesser curvature of the gastric angle and the gastric antrum. However, in this case, the lesion was found in an atypical location, particularly in the gastric body. Studies have shown that gastrointestinal symptoms caused by COVID-19 often appear earlier than respiratory symptoms, which is consistent with the patient’s description (8). The patient did not have common factors contributing to acute gastric mucosal injury or infections from other pathogens. By ruling out other factors and considering the results of ACE2 immunohistochemistry, we suspect that the patient’s acute gastric mucosal injury was caused by COVID-19 infection. After receiving treatment for COVID-19, the patient’s symptoms improved, and the gastric mucosal injury healed, supporting our hypothesis. Public perception predominantly associates COVID-19 infection with respiratory symptoms. Prior to treatment, the patient in this case did not believe their gastrointestinal bleeding was caused by COVID-19. However, through subsequent clinical management and investigation, the patient gradually accepted this rare manifestation. This underscores the need for both the public and healthcare professionals to be vigilant regarding COVID-19 infections presenting with uncommon gastrointestinal symptoms as the initial clinical feature.

Gastrointestinal damage can occur in patients with COVID-19 due to elevated inflammation, hypercoagulability, and endothelial dysfunction (9). We hypothesize that the patient’s lesion may have two possible causes. The first cause is attributed to the cytotoxic effect of the virus on the gastric mucosa. However, this possibility seems unlikely. Research has indicated that COVID-19 enters cells via the ACE2 receptor, and ACE2 receptor expression is significantly higher in the small intestine than in the stomach. Consequently, any direct cytotoxic effect of the virus would be more prominent in the small intestine. We believe that the second cause is more plausible. Recent studies have shown that increased D-dimer levels are a typical feature in patients with COVID-19 infection and coagulopathy (10), as observed in this particular patient. This hypercoagulable state originates from viral invasion of endothelial cells, leading to increased release of von Willebrand factor (vWF), abnormal platelet activation, and ultimately the formation of microvascular thrombi. When these thrombi involve the gastric mucosal microcirculation, they induce localized ischemic injury. This pathological cascade culminates in endoscopic manifestations including mucosal erosions, ulcer formation, and even hemorrhagic necrosis (11). Based on this, we hypothesize that the gastrointestinal mucosal injury and bleeding in this patient could be attributed to microvessel thrombosis associated with coagulopathy induced by COVID-19 (12).

Gastrointestinal manifestations of COVID-19 are well-recognized, with 50.5% of patients reporting non-specific symptoms such as anorexia, diarrhea, nausea, or vomiting (8). Acute gastrointestinal hemorrhage is a rare clinical complication, with only sporadic cases reported. It has not been documented as an initial presentation. Reported cases predominantly involve atypical sites and are associated with poor outcomes (13, 14). This hemorrhagic presentation not only reflects direct gastrointestinal injury by COVID-19 but also reveals virally induced systemic pathophysiological alterations. Notably, some cases exhibit gastrointestinal symptoms preceding typical respiratory manifestations, increasing diagnostic uncertainty. Such atypical presentation necessitates heightened clinical vigilance—particularly when patients present with unexplained gastrointestinal bleeding accompanied by elevated D-dimer levels—prompting consideration of COVID-19 as an underlying etiology. Critically, the risk of gastrointestinal hemorrhage correlates positively with COVID-19 severity. As demonstrated by Jin et al. (15), the proportion of severe/critical cases among COVID-19 patients with gastrointestinal symptoms significantly exceeds that among asymptomatic counterparts. A large case series by Lin et al. (16) demonstrated that the detection of SARS-CoV-2 RNA in fecal samples does not necessarily correlate with an increase in gastrointestinal symptoms. However, the confirmation of SARS-CoV-2 in GI tissue specimens is associated with a worst prognosis. Consequently, patients with severe COVID-19—especially those exhibiting coagulopathy markers—warrant intensified surveillance for bleeding risk. Although rare, COVID-19-related gastrointestinal bleeding is clinically significant, serving as both a marker of disease severity and a sign of multiorgan involvement. Strategies to improve patient outcomes include early identification of high-risk individuals, careful management of anticoagulants, and timely endoscopic interventions. Clinicians managing COVID-19 must remain alert to gastrointestinal symptoms, especially in patients with coagulation issues or unusual digestive complaints. By collaborating across disciplines and tailoring treatment plans, we can effectively address this serious complication and deepen our understanding of COVID-19’s impact on multiple organs, ultimately enhancing our ability to manage future outbreaks of emerging infectious diseases.

## Conclusion

In this case, pathological analysis showed significant lymphocytic infiltration in the necrotic tissue, suggesting a correlation with viral infection. Immunohistochemical detection of ACE2 revealed that as the patient recovered from COVID-19, the expression of ACE2 in the gastric tissue also decreased. After the patient’s recovery from COVID-19, follow-up gastroscopy confirmed the healing of gastrointestinal lesions, thus validating the effectiveness of our treatment.

Currently, although the COVID-19 epidemic trend is gradually declining, many people are still infected with its variants. This remains an undeniable public health concern. Therefore, future research on COVID-19 should focus on its gastrointestinal symptoms and related diseases. Further research will undoubtedly enhance our understanding and provide information for evidence-based decision-making in public health policies.

## Data Availability

The original contributions presented in the study are included in the article/supplementary material, further inquiries can be directed to the corresponding authors.
